# Magnetic resonance detects metabolic changes associated with chemotherapy-induced apoptosis

**DOI:** 10.1038/sj.bjc.6690459

**Published:** 1999-06

**Authors:** S M Ronen, F DiStefano, C L McCoy, D Robertson, T A D Smith, N M Al-Saffar, J Titley, D C Cunningham, J R Griffiths, M O Leach, P A Clarke

**Affiliations:** Clinical Magnetic Resonance Research Group; CRC Centre for Cancer Therapeutics; Electron Microscopy Unit; Department of Nuclear Medicine, Institute of Cancer Research; Royal Marsden Hospital, Down Road, Sutton, Surrey SM2 5PT, UK; CRC Biomedical Magnetic Resonance Research Group, St George's Hospital Medical School, Cranmer Terrace, London SW17 0RE, UK

**Keywords:** apoptosis, magnetic resonance spectroscopy (MRS), chemotherapy, glycolysis, poly(ADP-ribose) polymerase (PARP)

## Abstract

Apoptosis was induced by treating L1210 leukaemia cells with mechlorethamine, and SW620 colorectal cells with doxorubicin. The onset and progression of apoptosis were monitored by assessing caspase activation, mitochondrial transmembrane potential, phosphatidylserine externalization, DNA fragmentation and cell morphology. In parallel, ^31^P magnetic resonance (MR) spectra of cell extracts were recorded. In L1210 cells, caspase activation was detected at 4 h. By 3 h, the MR spectra showed a steady decrease in NTP and NAD, and a significant build-up of fructose 1,6-bisphosphate (F-1,6-P) dihydroxyacetonephosphate and glycerol-3-phosphate, indicating modulation of glycolysis. Treatment with iodoacetate also induced a build-up of F-1,6-P, while preincubation with two poly(ADP-ribose) polymerase inhibitors, 3-aminobenzamide and nicotinamide, prevented the drop in NAD and the build-up of glycolytic intermediates. This suggested that our results were due to inhibition of glyceraldehyde-3-phosphate dehydrogenase, possibly as a consequence of NAD depletion following poly(ADP-ribose) polymerase activation. Doxorubicin treatment of the adherent SW620 cells caused cells committed to apoptosis to detach. F-1,6-P was observed in detached cells, but not in treated cells that remained attached. This indicated that our observations were not cell line- or treatment-specific, but were correlated with the appearance of apoptotic cells following drug treatment. The ^31^P MR spectrum of tumours responding to chemotherapy could be modulated by similar effects. © 1999 Cancer Research Campaign


*British Journal of Cancer* (1999) **80**(7), 1035–1041
© 1999 Cancer Research Campaign
Article no. bjoc.1998.0459

### Magnetic resonance detects metabolic changes associated with chemotherapy-induced apoptosis

**SM Ronen[1], F DiStefano[2], CL McCoy[6], D Robertson[3], TAD Smith[4], NM Al-Saffar[1], J Titley[2], DC Cunningham[5], JR Griffiths[6], MO Leach[1] and PA Clarke[2]**

Cancer Research Campaign (CRC), [1]Clinical Magnetic Resonance Research Group, [2]CRC Centre for Cancer Therapeutics, [3]Electron Microscopy Unit, [4]Department of Nuclear Medicine, Institute of Cancer Research, [5]Royal Marsden Hospital, Down Road, Sutton, Surrey SM2 5PT, UK; [6]CRC Biomedical Magnetic Resonance Research Group, St George's Hospital Medical School, Cranmer Terrace, London SW17 0RE, UK

**Summary** Apoptosis was induced by treating L1210 leukaemia cells with mechlorethamine, and SW620 colorectal cells with doxorubicin. The onset and progression of apoptosis were monitored by assessing caspase activation, mitochondrial transmembrane potential, phosphatidylserine externalization, DNA fragmentation and cell morphology. In parallel, $^{31}$P magnetic resonance (MR) spectra of cell extracts were recorded. In L1210 cells, caspase activation was detected at 4 h. By 3 h, the MR spectra showed a steady decrease in NTP and NAD, and a significant build-up of fructose 1,6-bisphosphate (F-1,6-P) dihydroxyacetonephosphate and glycerol-3-phosphate, indicating modulation of glycolysis. Treatment with iodoacetate also induced a build-up of F-1,6-P, while preincubation with two poly(ADP-ribose) polymerase inhibitors, 3-aminobenzamide and nicotinamide, prevented the drop in NAD and the build-up of glycolytic intermediates. This suggested that our results were due to inhibition of glyceraldehyde-3-phosphate dehydrogenase, possibly as a consequence of NAD depletion following poly(ADP-ribose) polymerase activation. Doxorubicin treatment of the adherent SW620 cells caused cells committed to apoptosis to detach. F-1,6-P was observed in detached cells, but not in treated cells that remained attached. This indicated that our observations were not cell line- or treatment-specific, but were correlated with the appearance of apoptotic cells following drug treatment. The $^{31}$P MR spectrum of tumours responding to chemotherapy could be modulated by similar effects.

**Keywords:** apoptosis; magnetic resonance spectroscopy (MRS); chemotherapy; glycolysis; poly(ADP-ribose) polymerase (PARP)

Apoptosis is an active process of cell death characterized by distinct morphological and molecular changes which can be induced by many varied stimuli, including chemotherapeutic treatment of tumour cells (Hickman, 1992). A recent study of breast tru-cut tumour biopsies has demonstrated a good correlation between the detection of increased apoptosis early following chemotherapy and subsequent response to treatment (Ellis et al, 1997). Furthermore, the response of tumours to chemotherapy can be influenced by the expression of genes controlling the apoptotic process (White, 1996): in diffuse large cell lymphoma, for example, expression of the apoptosis inhibitor bcl-2, has been correlated with a poor prognosis (Hill et al, 1996). Thus the ability to detect or monitor apoptosis may be an important factor in planning patient care.

Magnetic resonance spectroscopy (MRS) is a technique that yields metabolic and bioenergetic information from cells or tissues in a dynamic, non-invasive and non-destructive manner. It can therefore be used for monitoring tumour progression or response to treatment. In this context, the identification of MR-detectable signals that are correlated with programmed cell death is important. In vivo $^{31}$P MRS studies of apoptosis have been limited, with preliminary reports describing a significant decrease in the phospholipid precursor phosphoethanolamine in non-Hodgkin's lymphoma patients who responded to treatment (Negendank et al, 1996). Using $^{31}$P MRS in vitro, a decrease in the phosphomonoester peak, composed of phosphoethanolamine and phosphocholine, was reported in dexamethasone-treated leukaemia lines (Adebodun and Post, 1994), while others have reported an increase in phosphocholine signal following tumour necrosis factor (TNF)-induced apoptosis in neutrophils (Nunn et al, 1996). In the leukaemia cells a steady decrease in nucleoside triphosphate (NTP) following genotoxic damage was detected (Adebodun and Post, 1994), whereas in doxorubicin-treated breast cancer cells this was preceded by a transient increase in adenosine 5′-triphosphate (ATP) (Neeman et al, 1990).

In light of the diversity of reported metabolic changes observed by MRS, we set out to investigate the temporal relationship between $^{31}$P MR-detected metabolic changes and some of the biological indicators of apoptosis. Our goal was to identify possible changes in the MRS spectra of apoptosing cells and to correlate these changes with known events leading to cell death following chemotherapeutic treatment. Here, we describe $^{31}$P MRS data obtained both from L1210 murine lymphocytic leukaemia cells treated with the alkylating nitrogen mustard mechlorethamine (HN2) and from SW620 human colorectal cancer cells treated with the anthracyclin doxorubicin. Despite the differences between the two model systems, we show similar MRS-detectable signals from cells dying by apoptosis. We demonstrate that, in L1210 cells, these MRS signals occur relatively early in the apoptotic process, and propose a possible mechanism explaining our MR observations.

*Received 1 October 1998*
*Revised 24 November 1998*
*Accepted 27 November 1998*

*Correspondence to:* SM Ronen

### MATERIALS AND METHODS

Reagents were obtained from Sigma or Gibco UK unless otherwise specified.

### Cell culture and treatment

L1210 cells were cultured in suspension in RPMI-1640 supplemented with 10% horse serum, 2 mM L-glutamine, 50 U $ml^{-1}$ penicillin and 50 μg $ml^{-1}$ streptomycin in 5% carbon dioxide. SW620 cells were cultured in Dulbecco's modified Eagle's medium (DMEM) supplemented with 10% fetal calf serum (FCS), with other conditions as above. Cell viability was determined by trypan blue exclusion.

For investigations of apoptosis, L1210 cells were treated with 50 μM HN2 and SW620 cells were treated with 1 μM doxorubicin. Cells were treated by replacing the normal growth medium with medium supplemented with the appropriate drug. To inhibit glyceraldehyde-3-phosphate dehydrogenase (GAPDH) cells were treated with 250 μM iodoacetate. Protein kinase C activity was inhibited by pre-incubating for 1 h with 100 nM staurosporine. Poly(ADP-ribose)polymerase (PARP) activity was blocked by pre-incubating for 1 h with 15 mM nicotinamide or 10 mM 3-aminobenzamide (Biomol).

### Morphology

A total of $2 \times 10^6$ cells were fixed in 2% glutaraldehyde in 0.05 M phosphate buffer plus 0.05 sucrose, pH 7.3 overnight at 4°C. Samples were post-fixed in 1% osmium tetroxide, dehydrated, infiltrated and embedded in Epon. One-micrometer sections were stained with toluidine blue.

### FITC–annexin-V staining

Cells ($1 \times 10^6$) were resuspended in binding buffer (HEPES-buffered saline, 5 mM calcium chloride), incubated at 37°C for 15 min with 1 μg $ml^{-1}$ fluorescein isothiocyanate (FITC)–annexin-V (R&D Systems) and 5 μg $ml^{-1}$ propidium iodide, and analysed by Fluorescence Activated Cell Sorting (FACS, Ortho Clinical Diagnostics flow cytometer) to determine cell size, annexin binding and propidium iodide exclusion.

### Cell cycle analysis

Cells ($1 \times 10^6$) were fixed with 70% ethanol for 30 min at 4°C, incubated with 100 μg $ml^{-1}$ RNAase and 40 μg $ml^{-1}$ propidium iodide in phosphate-buffered saline (PBS) for 30 min at 37°C. DNA histograms were generated by FACS analysis.

### 3,3′-dihexyloxacarbocyanine iodide (DiOC6(3)) staining

DiOC6(3) staining was used to determine mitochondrial transmembrane potential as previously described (Zamzami et al, 1995). A suspension of $1 \times 10^6$ cells $ml^{-1}$ was incubated at 37°C for 30 min with 50 nM DiOC6(3) and then immediately analysed by FACS.

### Caspase activity

Caspase activity (DEVDase) was determined using a commercial colorimetric assay (Clontech, USA). Extracts of $2 \times 10^6$ cells were incubated for 60 min with aspartate–glutamate–valine–aspartate–ρ–nitroanilide (DEVD-ρ-NA). ρ-nitroanilide release following caspase activation was measured by absorbance at 400 nM (Shimadzu spectrophotometer). Background absorbance was calculated from controls incubated in the absence of substrate.

### Magnetic resonance spectroscopy

One to $2 \times 10^8$ control and treated cells were extracted using a modification of the dual-phase extraction method (Ronen et al, 1990; Tyagi et al, 1996). Briefly, a pellet of saline-rinsed L1210 cells or detached SW620 cells was vortexed in 10 ml of ice-cold methanol, followed by 10 ml of ice-cold chloroform and 10 ml ice-cold deionized water. For adherent SW620 cells, cells were rinsed, covered with ice-cold methanol, scraped off the flask surface, collected and treated as above. Following phase separation and solvent removal, the water phase was resuspended in $D_2O$ ('heavy water', deuterium oxide) with 10 mM EDTA at pH 8.2 and the lipid phase was resuspended in $CDCl_3$ supplemented with methanolic EDTA. Samples were stored at –20°C. MRS spectra were acquired at room temperature on a 250 MHz Bruker spectrometer at 101 MHz using a 90° flip angle, a 7 s relaxation delay and broad-band proton decoupling during acquisition. Metabolite contents were determined by integration, normalized relative to an external methylenediphosphonic acid (MDPA) reference peak and corrected for the number of live cells extracted. NTP content was calculated from the average of βNTP and γNTP peaks, and nucleoside diphosphate (NDP) content from βNDP and αNDP. Due to partial overlap of nicotinamide-adenine dinucleotide (NAD) and αNTP peaks, NAD/H content was determined by measuring the total integral and subtracting the contribution of NTP. To identify the new peaks observed in the spectra of apoptosing cells, spectra of candidate metabolites were recorded under identical conditions as the extracts. Assignments were confirmed by spiking two treated cell extracts at pH 8.2 and another two extracts at pH 6.2. Following treatment, in order to separate the overlapping phosphoethanolamine and fructose 1,6-bisphosphate (6-P) peaks, the extract pH was acidified from 8.2 to 6.2 using hydrochloric acid (10 mM).

Results are expressed as mean ± s.e.m. and $n \geq 3$ unless otherwise specified. Statistics were performed using a Student's standard *t*-test and differences were assumed to be significant when $P < 0.05$.

### RESULTS

To monitor the progression of apoptosis in L1210 leukaemia cells treated with 50 μM HN2, different biological markers were monitored over time (Figure 1). We assessed indicators that are associated with the end stage execution step of apoptosis (Figure 1A) including DNA fragmentation, phosphatidylserine externalization, cell size and morphological changes (not shown), as well as indicators of the earlier stages of the apoptotic process, namely caspase activation (DEVDase) and loss of mitochondrial transmembrane potential (Figure 1B). As illustrated in Figure 1B, we were able to detect early signs of apoptosis at 4 h following HN2 treatment with the increase in caspase activity to 340% representing the most significant change at that time.

Extracts from control and HN2-treated L1210 cells were prepared using a chloroform:methanol:water extract. Representative spectra obtained from untreated control cells and from cells treated for 3 h with HN2 are shown in Figure 2. The extract from control cells

*© 1999 Cancer Research Campaign*

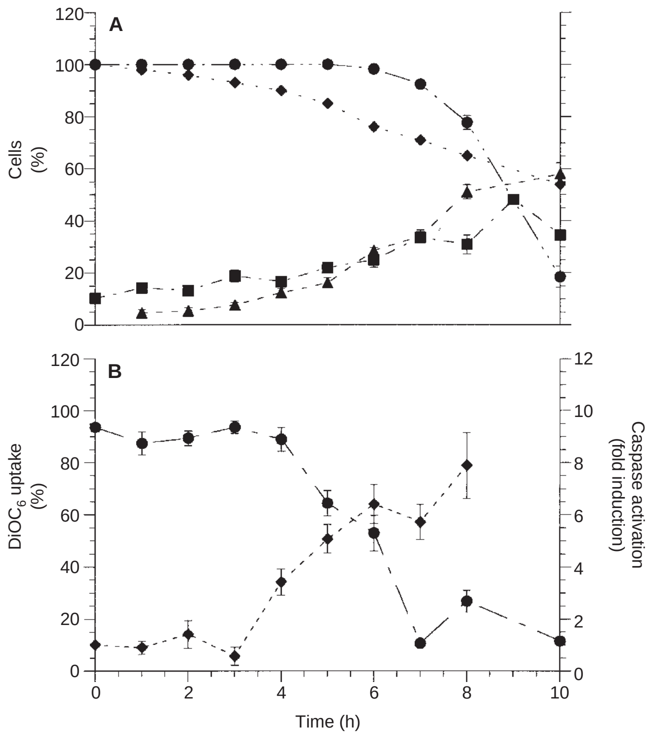

**Figure 1** Time course for 50 μM HN2-treated L1210 cells of (**A**): (●) cell viability, (△) presence of hypodiploid cells, (◆) cell size, and (■) phosphatidylserine externalization. (**B**): (●) $DiOC_6$ uptake and (◆) caspase activation

(Figure 2A) showed a similar metabolic profile to previously published data from L1210 extracts (Berners-Price et al, 1991). Following-HN2 treatment (Figure 2B), a decrease in nucleotide signals was observed, as well as the build-up of two prominent peaks at 4.3 and 4.4 ppm and two smaller peaks at 4.9 and 5.4 ppm. The identity of the peaks was initially examined by recording $^{31}$P spectra of candidate compounds under the same conditions as the extracts. The peaks were tentatively identified as fructose 1,6-bisphosphate (F-1,6-P) at 4.3 (6-P) and 4.4 (1-P) ppm, glycerol-3-phosphate (G-3-P) at 4.9 ppm and dihydroxyacetone phosphate (DHAP) at 5.4 ppm. This was confirmed by duplicate experiments recording a $^{31}$P spectrum from an extract of apoptotic cells, spiking the extract with candidate compounds and confirming that the areas of the new peaks had increased following spiking. Further confirmation was obtained by acidifying two other extracts to pH 6.2 and spiking again (at pH 6.2 F-1,6-P resonated at 2.2 and 2.4 ppm, G-3-P resonated at 2.6 ppm and DHAP at 3.5 ppm).

Figure 2 demonstrates that the increase in F-1,6-P following HN2 treatment of L1210 cells was very significant, rising from below detection level (ca. 0.5 fmol cell$^{-1}$) to a maximum of 30 ± 6 fmol cell$^{-1}$ following 3 h of treatment. G-3-P and DHAP content was approximately 20% that of F-1,6-P. The time course for the appearance of F-1,6-P, DHAP and G-3-P is illustrated in Figure 3A ($n$ = 3–5). F-1,6-P reached a maximum level 3 h post treatment and declined by 6 h. Interestingly, F-1,6-P, DHAP and G-3-P could be detected slightly earlier than caspase activation.

As mentioned above there was a decrease in nucleotide content during apoptosis induced by HN2. The decrease in both NTP+NDP and NAD was observed within the first hour following drug treatment, with the nucleotides steadily decreasing to below detection level at 6 h post treatment ($P$ < 0.01 following 3 h of treatment, Figure 3B). A plot of NTP content alone showed an identical trend.

The lipid phase of the cell extracts includes the phosphorous-containing membrane components of the cells and in particular phosphatidylcholine and sphingomyelin whose degradation might be involved in signalling during apoptosis. However, the $^{31}$P spectra obtained from the chloroform phase of HN2-treated cells (data not shown) demonstrated no significant differences from controls. The phospholipid precursors, phosphocholine and phosphoethanolamine, which could be detected in the water-soluble fraction of the extract (Figure 2) decreased slightly during the first 2 h of treatment and then remained unchanged at 60% ± 20% of control ($n$ = 3–5, $P$ < 0.05 for 2 h and $P$ < 0.07 for 3–6 h).

To confirm that our observations were not specific to cell line or treatment we investigated a second model system. To also confirm that the MR results were not simply due to cells sustaining drug-induced DNA damage but were related to the presence of cells destined to die, we chose the adherent epithelial SW620 colorectal cell line. Apoptosis was induced by treating with 1 μM doxorubicin. Morphological observation (Figure 4 A, B) indicated that apoptosis was ultimately detected almost exclusively in the cells that detached from the surface of the culture flask following treatment and floated in the medium, consistent with earlier studies of SW620 cells (Hague et al, 1993; Heerdt et al, 1994). This was confirmed by cell cycle analysis: the presence of hypodiploid cells was detected exclusively in the population of floating cells. In duplicate analysis, 20–30% of the floating cells were apoptotic at 8 h, 50–60% at 16 h, and after 24 h of doxorubicin treatment all of the floating SW620 cells were hypodiploid. Figure 4 also illustrates the spectra obtained from adherent control (Figure 4C), as well as adherent and floating cells following 24 h of doxorubicin treatment (Figure 4D and 4E respectively). In the attached treated cells, a 30–40% ($n$ = 2) decrease in NTP content was observed compared to controls. However, in contrast to L1210 cells treated with HN2, no new signals could be detected in the attached cell population following exposure to the chemotherapeutic agent. On the other hand, in the SW620 floating population, composed of cells committed to apoptosis, F-1,6-P could be detected by 8 h following treatment and was clearly visible at 24 h (Figure 4E). In the floating cells the NTP content had decreased to zero after 24 h of doxorubicin treatment, and a drop in glycerophosphocholine was also noted. The presence of a significant amount of UDP-sugars resonating at a similar chemical shift to NAD prevented the estimation of changes in NAD.

The appearance of F-1,6-P, DHAP and G-3-P following treatment of L1210 and SW620 cells with agents that result in DNA damage implied that glycolysis was being modulated. A number of explanations were possible including inhibition of GAPDH or activation of F-1,6-P synthesis. F-1,6-P synthesis by phosphofructokinase is regulated by protein kinase C (Eigenbrodt et al, 1994). The role of protein kinase C activation was examined by preincubating the cells, prior to application of the apoptotic stimulus, with 100 nM staurosporine (STS), a protein kinase inhibitor with a broad spectrum of activity (e.g. Mueller et al, 1995). This dose of STS induced cytostasis, but was not cytotoxic. Following preincubation with STS, HN2-treated L1210 cells showed the same increase in glycolytic intermediates as cells treated with HN2 alone (Table 1). Cells treated with STS alone produced a spectrum identical to controls.

© 1999 Cancer Research Campaign

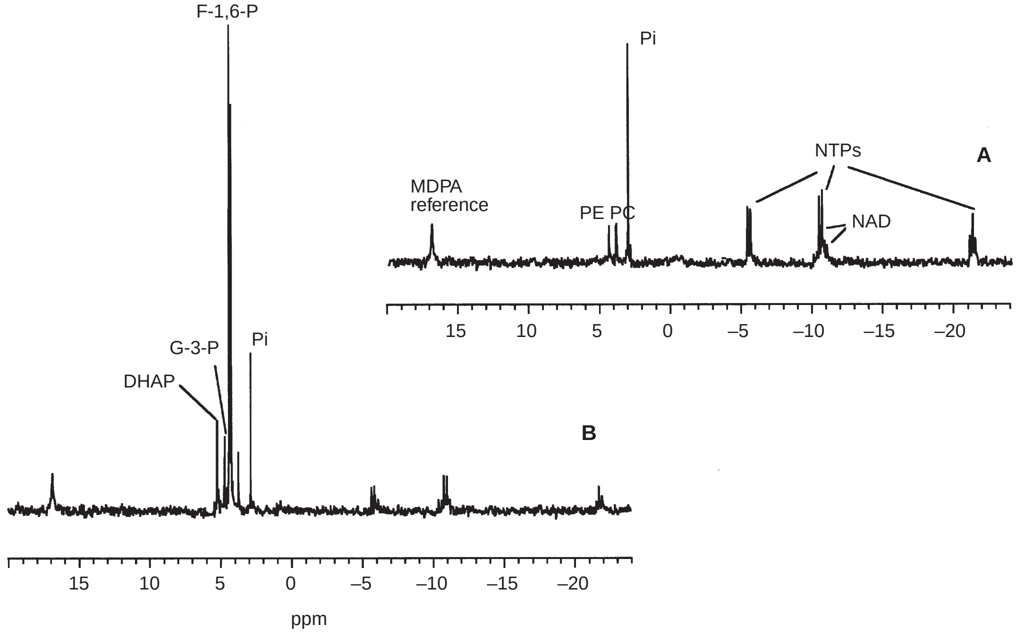

**Figure 2** [1]H decoupled [31]P MRS spectra of extracts from (**A**) control L1210 cells, (**B**) 3 h treated L1210 cells following 3 h of treatment with 50 µM HN2. Spectra are the result of 10 000 scans plotted with a line broadening of 0.5 Hz

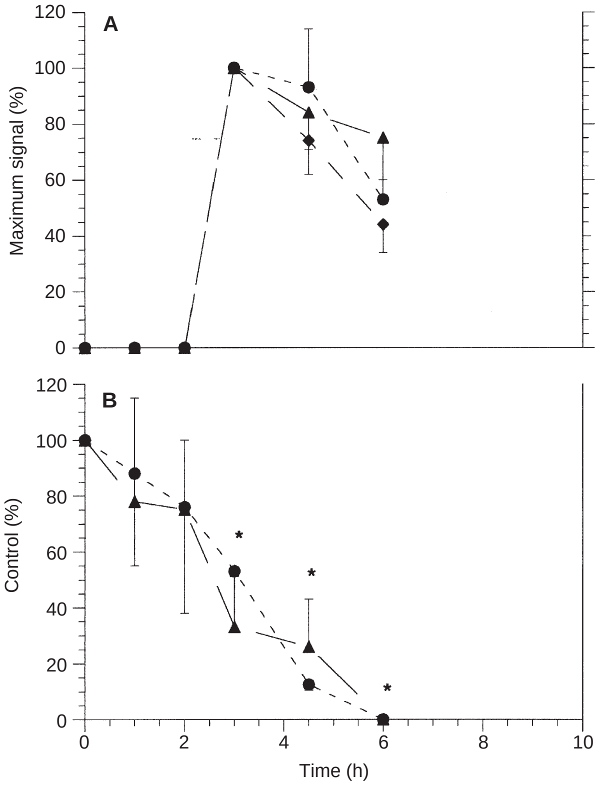

**Figure 3** Time course for 50 µM HN2-treated L1210 cells of (**A**): (●) F-1,6-P, (◆) DHAP, (▲) G-3-P, as detected by MRS and expressed relative to the average maximum value detected 3 hours post treatment. (**B**): (●) NTP+NDP, (▲) NAD, as detected by MRS. Error bars represent s.e.m. ($n \geq 3$). *Indicates a statistically significant difference between treated and control and *P*-values were as follows. For NTP+NDP: at 3 h $P = 0.0005$; at 4.5 h $P = 0.0001$; at 6 h $P = 0.0001$. For NAD: at 3 h $P = 0.01$; at 4.5 h $P = 0.004$; at 6 h $P = 0.0001$

To test the hypothesis that the appearance of F-1,6-P, DHAP and G-3-P was due to inhibition of GAPDH, we used iodoacetate, shown to specifically inhibit GAPDH at concentrations below 1 mM (Webb, 1966). We treated L1210 cells with 250 µM iodoacetate, a dose that had minimal effects on viability (> 95% at 24 h). Following 4 and 8 h of treatment, F-1,6-P signals representing 3 and 6 fmol $cell^{-1}$, respectively, could be observed in the cell extracts, while NTP content dropped to 80% of control (Table 1). G-3-P and DHAP remained below detection level at 4 h and were on the order of 1 fmol $cell^{-1}$ at 8 h. This indicated inhibition of GAPDH.

As mentioned above, NAD levels decreased within an hour following HN2 treatment of L1210 cells. In addition to being a cofactor required for GAPDH activity, NAD is also a substrate for PARP, an enzyme involved in DNA damage repair (Satoh and Lindahl, 1992). DNA damage induced by either HN2 or doxorubicin could result in PARP activation, leading to the consumption of NAD, and NAD depletion could in turn result in inhibition of GAPDH. To test this hypothesis we pretreated the cells with 10 mM 3-aminobenzamide (3-ABA), a competitive inhibitor of PARP. One hour preincubation of L1210 cells with 10 mM 3-ABA effectively blocked the cytotoxic effects of HN2 at 24 h, increasing cell viability from 9% to 82% relative to untreated control ($P < 0.05$) and preventing the appearance of hypodiploid cells as evidenced by FACS. The inhibitor concentration used was based on previous work (e.g. Milam and Cleaver, 1984), and was assessed in our system by determining the effects of 1, 5, 10 and 15 mM 3-ABA. The minimum dose of 3-ABA required to significantly block the effect of HN2 was 10 mM. Importantly, preincubation with 10 mM 3-ABA was found to prevent the build-up of F-1,6-P, DHAP and G-3-P, as well as the decrease in NAD (Table 1). To try and rule out possible non-specific effects of 3-ABA we also investigated a second PARP inhibitor, nicotinamide, the by product of ADP-ribose polymerization. Similar to our observations with 3-ABA, pretreatment with 15 mM nicotinamide also blocked

 © 1999 Cancer Research Campaign

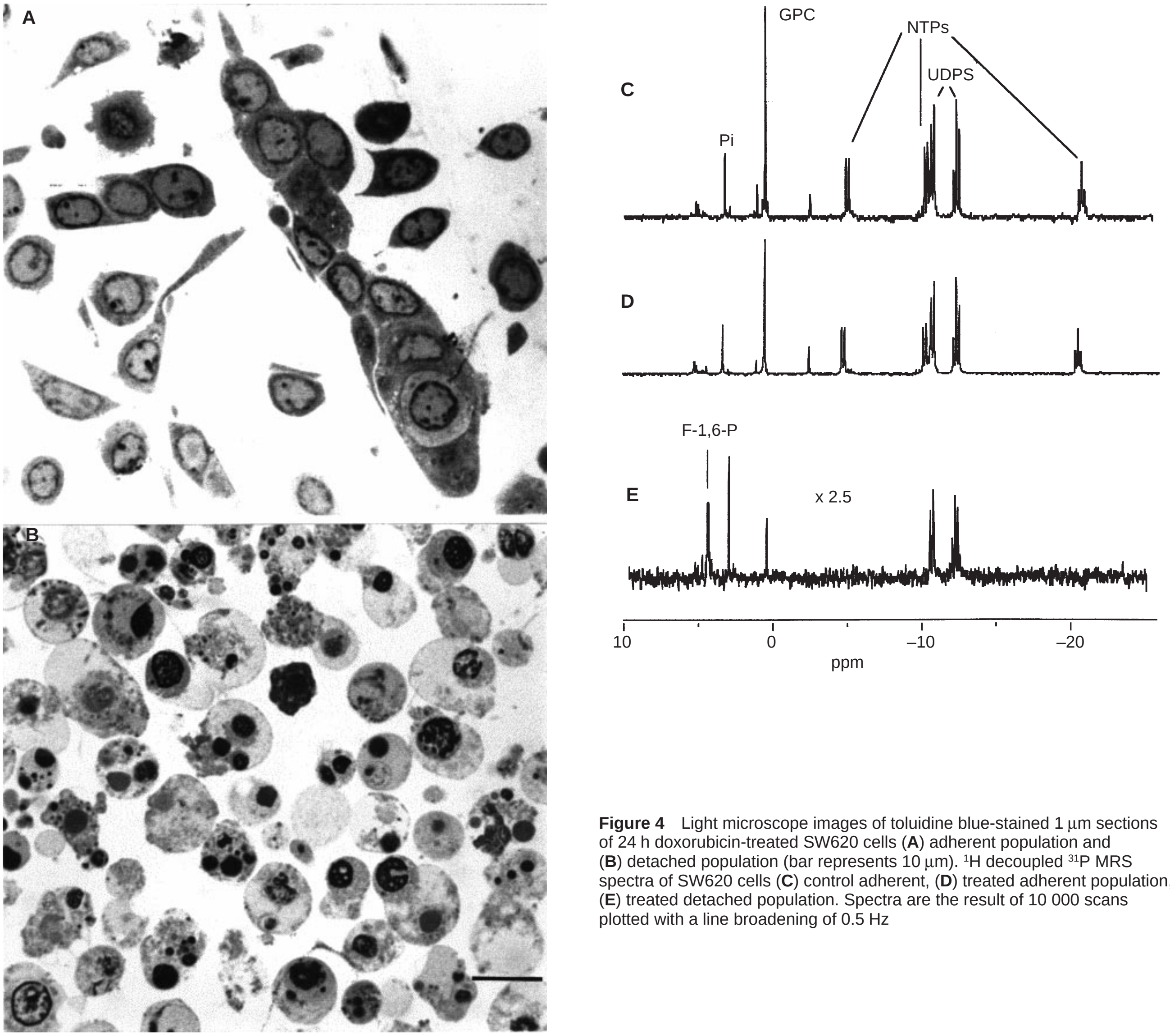

**Figure 4** Light microscope images of toluidine blue-stained 1 µm sections of 24 h doxorubicin-treated SW620 cells (**A**) adherent population and (**B**) detached population (bar represents 10 µm). $^1$H decoupled $^{31}$P MRS spectra of SW620 cells (**C**) control adherent, (**D**) treated adherent population, (**E**) treated detached population. Spectra are the result of 10 000 scans plotted with a line broadening of 0.5 Hz

**Table 1** Summary of MRS-detected metabolic changes in L1210 cells following treatment

| Treatment | Glycolytic intermediates (a) | NAD (b) | *n* |
|---|---|---|---|
| HN2 (3 h) | ⇑ | ⇓ | 6 |
| I-acetate | ↑ | ↓ | 4 |
| STS + HN2 | ⇑ | ⇓ | 2 |
| STS | → | → | 2 |
| Nic + HN2 | → | ↑ | 4 |
| Nic | → | ↑ | 1 |
| 3-ABA + HN2 | → | → | 2 |
| 3-ABA | → | → | 1 |

→ unchanged relative to untreated control; (a) ⇑ observed F-1,6-P, G-3-P and DHAP, ↑ observed F-1,6-P; (b) ↓ down to 60–80% of control, ⇓ down to 0% of control, ↑ up 150–200% of control. HN2: mechlorethamine; STS: staurosporine; Nic: nicotinamide; 3-ABA: 3-aminobenzamide.

the appearance of the glycolytic intermediates and prevented the decrease in NAD in the HN2-treated L1210 cells.

### DISCUSSION

In this study, we have used $^{31}$P MRS to monitor metabolic changes which occur in vitro in tumour cells following apoptosis induced by chemotherapeutic treatment. We have identified the MRS signals associated with apoptosis in our cells and have compared the temporal evolution of these signals with the appearance of a number of known biological markers of apoptosis. In addition, we have tried to understand the mechanism responsible for the MR-detectable metabolic changes. The importance of such studies is twofold: first they can aid in interpreting MRS changes observed in treated tumours, and second they can contribute to a further understanding of some of the steps involved in apoptotic cell death.

Compared to in vitro systems, the number of cells synchronously apoptosing in vivo can be small (Ellis et al, 1997), and

© 1999 Cancer Research Campaign 

consequently MRS changes might be difficult to detect and interpret without prior knowledge of the likely spectral changes. Furthermore, the precise identification of new peaks observed in an in vivo spectrum can often remain uncertain. To avoid these problems we concentrated on a well-defined model system in which a relatively large number of cells were induced to undergo apoptosis simultaneously. In addition, we performed our investigations on cell extracts, and thus the high spectral resolution and the ability to manipulate the samples enabled us to unambiguously identify the MRS changes occurring in apoptosing cells.

Following HN2-treatment, all the L1210 cells ultimately died by apoptosis. However, at any given time point, it was difficult to distinguish between cells that had already committed to death and those that had not. This prevented us addressing the question whether our MRS observations were a consequence simply of DNA damage or were correlated with cell death. We therefore also investigated the adherent SW620 cells. In contrast to L1210 cells, SW620 cells could be separated into adherent cells and cells that detached from the culture flask following chemotherapeutic treatment. Only the cells that detached ultimately showed morphological signs of apoptosis and DNA fragmentation, making it possible to isolate cells which had committed to apoptosis. The substantial build-up of glycolytic intermediates observed in detached SW620 cells and treated L1210 cells, but not in adherent treated SW620 cells, lead us to conclude that the MRS changes were correlated with commitment to apoptosis. Furthermore, SW620 data also indicated that our observations were not specific to L1210 cells or HN2-treatment.

The MR detectable changes in NTP, NAD and the glycolytic intermediates occurred relatively early, with changes in the metabolic profile of L1210 cells treated with HN2 apparent within 2–3 h following treatment. The increase in glycolytic intermediates could not simply be ascribed to a small number of apoptotic cells appearing at 3 h, since the concentrations of glycolytic intermediates did not continue to rise after 3 h when the majority of apoptotic cells appeared.

NTP decreased steadily following HN2 treatment of L1210 cells. At 3 h, NTP content was 53% of control and dropped to 12% at 4.5 h and 0% at 6 h (Figure 3B). During this time, plasma membrane integrity remained at > 98% (Figure 1A). Cell size decreased to 93% of control at 3 h post treatment, 90% at 4 h, and 76% at 6 h (Figure 1A). Thus, the decrease in cellular energy content was not due to leakage as a result of damaged plasma membranes, and could not be explained entirely by cell shrinkage. HPLC analysis of these cells has shown that over 60% of NTP in these cells is composed of ATP (Berners-Price et al, 1991). The drop in NTP content in our cells is therefore most likely to be due to energy utilization by the cell for apoptosis and is consistent with earlier reports (Eguchi et al, 1997).

We considered a number of mechanisms to explain the build-up of glycolytic intermediates in our cells. SW620 cells have a mutant, non-functional, p53 gene product (O'Connor et al, 1997), ruling out p53 involvement. One possible explanation of our results was activation of F-1,6-P synthesis by protein kinase C, which activates 6-phosphofructo-2-kinase, leading to increased synthesis of fructose 2,6 bisphosphate, an activator of phosphofructokinase (Eigenbrodt et al, 1994). However, preincubation of L1210 cells with the protein kinase inhibitor staurosporine prior to HN2 treatment did not prevent F-1,6-P, DHAP and G-3-P build-up, which were detected at a level comparable to that observed with HN2 alone. This ruled out involvement of phosphorylation by protein kinase C in explaining our results.

A more likely explanation for the build-up of F-1,6-P together with DHAP and G-3-P is inhibition of the flux through the glycolytic pathway at the level of GAPDH. Inhibition of GAPDH would lead to build-up of glyceraldehyde-3-phosphate and DHAP. The absence of a detectable glyceraldehyde-3-phosphate peak is most likely a consequence of the isomerase reaction equilibrium. Typically, 90% of the triose mixture is present as DHAP and 10% as glyceraldehyde-3-phosphate (White et al, 1978). Based on the concentration of DHAP detected in our experiments (up to 6 fmol $cell^{-1}$), the maximum possible glyceraldehyde-3-phosphate concentration would therefore be 0.66 fmol $cell^{-1}$. This was at the limit of MRS detection level in our system. The equilibrium constant for the aldolase favours, in the absence of DHAP and glyceraldehyde-3-phosphate clearance, the synthesis of F-1,6-P (White et al, 1978), explaining the presence of a relatively high F-1,6-P concentration. Following iodoacetate treatment, a F-1,6-P peak could be detected in the cell extracts, and although the intensity of the F-1,6-P peak was not as large as that detected following HN2-treatment, its appearance did nonetheless indicate that inhibition of glycolysis at the level of GAPDH was a possible explanation of our results.

A possible explanation of GAPDH inhibition and the subsequent accumulation of glycolytic intermediates was NAD depletion. A reduction in NAD has been reported to inhibit GAPDH as well as the whole of the glycolytic pathway, with F-1,6-P accumulating in the cell (Eigenbrodt et al, 1994). The decrease in NAD observed following HN2 treatment could be due to activation of PARP, which consumes NAD. A substantial drop in NAD levels, as well as a depletion in ATP following PARP activation, have been reported in other systems (Satoh and Lindahl, 1992; Martin et al, 1997). We tested this hypothesis by pre-incubating L1210 cells with two different PARP inhibitors: 3-ABA, and nicotinamide. Both inhibitors prevented NAD depletion in our cells and no accumulation of F-1,6-P, G-3-P or DHAP was observed in either case. PARP inhibition also blocked the cytotoxic effects of HN2 for at least 24 hours, suggesting a possible link between NAD depletion, accumulation of glycolytic intermediates and cell death induced by DNA damage (Nosseri et al, 1994; Zhang et al, 1994). Furthermore, PARP is proteolytically degraded by caspase 3, but in our model, the decrease of NAD was observed as early as 1 h following treatment and well before caspase activation was apparent (4 h). Nonetheless, one has to treat this explanation with caution. Apoptosis can occur in the absence of PARP and in some cases PARP-deficient cells are more sensitive to DNA damage than wild-type cells (e.g. Lerst et al, 1997). In addition, PARP is a member of a gene superfamily which are all NAD-metabolizing enzymes (Koch-Nolte and Haag, 1997) and we can not rule out the involvement of another such enzyme in explaining our results.

In conclusion, we have shown that, using $^{31}$P MRS, we detect an increase in the glycolytic intermediates F-1,6-P, DHAP and G-3-P in cells apoptosing following exposure to DNA damaging agents. This indicated an inhibition of the glycolytic pathway at the level of GAPDH and could be explained by NAD depletion by PARP. Interestingly, this inhibition occurred relatively early in the apoptotic cascade. We speculate that, when DNA damage is substantial enough to induce apoptosis, the $^{31}$P MRS spectrum of treated tumours could be affected early following treatment, particularly in the phosphomonoester region where F-1,6-P resonates. We will be extending our investigations to in vivo models to determine the possible value of MRS in assessing increased apoptosis in treated tumours.

*© 1999 Cancer Research Campaign*

### ACKNOWLEDGEMENTS

The authors would like to thank Dr DEV Wilman for his assistance in the use of the NMR spectrometer, and Prof P Workman and Dr M Stubbs for critically reviewing this manuscript. CRC funding is gratefully acknowledged by SMR and MOL (grant number SP SP1780/0103), JRG and CLM (grant number SP 1971/0402) and PAC (grant number SP2330).

### REFERENCES

Adebodun F and Post JF (1994) $^{31}$P NMR characterization of cellular metabolism during dexamethasone induced apoptosis in human leukemic cell lines. *J Cell Physiol* **158**: 180–186

Berners-Price-SJ, Sant ME, Christopherson RI and Kuchel PW (1991) $^{1}$H and $^{31}$P NMR and HPLC studies of mouse L1210 leukemia cell extracts: the effect of Au(I) and Cu(I) diphosphine complexes on the cell metabolism. *Magn Reson Med* **18**: 142–158

Eguchi Y, Shimizu S and Tsujimoto Y (1997) Intracellular ATP levels determine cell death fate by apoptosis or necrosis. *Cancer Res* **57**: 1835–1840

Eigenbrodt E, Gerbracht U, Mazurek S, Presket P and Friis R (1994) Carbohydrate metabolism and neoplasia: new perspectives for diagnosis and therapy. In *Biochemical and Molecular Aspects of Selected Cancers*, Prestlow TG and Prestlow TP (eds), pp. 311–385. Academic Press, San Diego

Ellis PA, Smith IE, McCarthy K, Detre S, Salter J and Dowsett M (1997) Preoperative chemotherapy induces apoptosis in early breast cancer [letter]. *Lancet* **349**: 849

Hague A, Manning AM, Hanlon KA, Huschtscha LI, Hart D and Paraskeva C (1993) Sodium butyrate induces apoptosis in human colonic tumour cell lines in a p-53 independent pathway: implications for the possible role of dietary fibre in the prevention of large-bowel cancer. *Int J Cancer* **55**: 498–505

Heerdt BG, Houston MA and Augenlich LH (1994) Potentiation by specific short-chain fatty acids of differentiation and apoptosis in human colonic carcinoma cell lines. *Cancer Res* **54**: 3288–3294

Hickman JA (1992) Apoptosis induced by anticancer drugs. *Cancer Metastasis Rev* **11**: 121–139

Hill ME, MacLennan KA, Cunningham DC, Vaughan-Hudson B, Burke M, Clarke P, Di-Stefano F, Anderson L, Vaughan-Hudson G, Mason D, Selby P and Linch DC (1996) Prognostic significance of bcl-2 expression and bcl-2 major breakpoint region rearrangement in diffuse large cell non-Hodgkin’s lymphoma: a British National Lymphoma Investigation Study. *Blood* **88**: 1046–1051

Koch-Nolte F and Haag F (1997) Mono(ADP-ribosyl)transferases and related enzymes in animal tissues. Emerging gene families. *Adv Exp Med Biol* **419**: 1–13

Leist M, Single B, Kunstle G, Volbracht C, Hentze H and Nicotera P (1997) Apoptosis in the absence of poly-(ADP-ribose) polymerase. *Biochem Biophys Res Commun* **233**: 518–522

Martin DS, Stolfi RL and Colofiore JR (1997) Perspective: the chemotherapeutic relevance of apoptosis and a proposed biochemical cascade for chemotherapeutically induced apoptosis. *Cancer Invest* **15**: 372–381

Milam KM and Cleaver JE (1984) Inhibitors of poly(adenosine diphosphate-ribose) synthesis: effect on other metabolic processes. *Science* **223**: 589–591

Mueller SG, Schraw WP and Richmond A (1995) Activation of protein kinase C enhances the phosphorylation of the type B interleukin-8 receptor and stimulates its degradation in non-hematopoietic cells. *J Biol Chem* **270**: 10439–10448

Neeman M, Eldar H, Rushkin E and Degani H (1990) Chemotherapy-induced changes in the energetics of human breast cancer cells; $^{31}$P- and $^{13}$C-NMR studies. *Biochim Biophys Acta* **1052**: 255–263

Negendank W, Padavic Shaller K, Li CW, Murphy Boesch J, Schilder RS, Smith MR and Brown TR (1996) Phosphomonoester changes in vivo and treatment response in human non-Hodgkin’s lymphomas (meeting abstract). *Proc Annu Meet Am Assoc Cancer Res* **37**: A1343

Nosseri C, Coppola S and Ghibelli L (1994) Possible involvement of poly(ADP-ribosyl) polymerase in triggering stress-induced apoptosis. *Exp Cell Res* **212**: 367–373

Nunn AV, Barnard ML, Bhakoo K, Murray J, Chilvers EJ and Bell JD (1996) Characterisation of secondary metabolites associated with neutrophil apoptosis. *FEBS Lett* **392**: 295–298

O’Connor PM, Jackman J, Bae I, Myers TG, Fan S, Mutoh M, Scudiero DA, Monks A, Sausville EA, Weinstein JN, Friend S, Fornace AJ Jr and Kohn KW (1997) Characterization of the p53 tumor suppressor pathway in cell lines of the National Cancer Institute anticancer drug screen and correlations with the growth-inhibitory potency of 123 anticancer agents. *Cancer Res* **57**: 4285–4300

Ronen SM, Stier A and Degani H (1990) NMR studies of the lipid metabolism of T47D human breast cancer spheroids. *FEBS* **266**: 147–150

Satoh MS and Lindahl T (1992) Role of poly(ADP-ribose) formation in DNA repair. *Nature* **356**: 356–358

Tyagi RK, Azrad A, Degani H and Salomon Y (1996) Simultaneous extraction of cellular lipids and water-soluble metabolites: evaluation by NMR spectroscopy. *Magn Reson Med* **35**: 194–200

Webb JL (1966) Iodoacetate and iodoacetamide. In: *Enzyme and Metabolic Inhibitors*, pp. 1–270. Academic Press: New York

White A, Handler P, Smith EL, Hill RL and Lehman IR (1978) *Principles of Biochemistry*, 6th edn. McGraw-Hill Kogakusha: Tokyo

White E (1996) Life, death, and the pursuit of apoptosis. *Genes Dev* **10**: 1–15

Zamzami N, Marchetti P, Castedo M, Zanin C, Vayssiere J-L, Petit PX and Kroemer G (1995) Reduction in mitochondrial potential constitutes an early irreversible step of programmed lymphocyte death in vivo. *J Exp Med* **181**: 1661–1672

Zhang J, Dawson TM, Dawson VL and Snyder SH (1994) Nitric oxide activation of poly(ADP-ribose) synthetase in neurotoxicity. *Science* **263**: 687–689

© 1999 Cancer Research Campaign
